# Real-time digital assistance improves surgeon efficiency and autonomy in robotic microvascular anastomosis: a pilot feasibility study

**DOI:** 10.1007/s11701-026-03347-z

**Published:** 2026-04-02

**Authors:** Jesse Selber, Harishanker Jeyarajan, Ricardo Hanel

**Affiliations:** 1 Department of Plastic & Reconstructive Surgery, Corewell Health William Beaumont University Hospital, Royal Oak, MI USA; 2 Department of Otolaryngology – Head and Neck Surgery, University of Alabama at Birmingham, Birmingham, AL USA; 3 Department of Neurosurgery, Baptist Medical Center Jacksonville, Jacksonville, FL USA; 4 Department of Neurosurgery, University of Florida College of Medicine, Jacksonville, USA

**Keywords:** Robotic microsurgery, Digital assistance platform, Microvascular anastomosis, Surgical workflow efficiency, Microsurgery, Digital surgery

## Abstract

Robotic microsurgery offers greater precision compared to manual surgical techniques and enables vascular anastomoses at submillimeter scales. This pilot study evaluated the incorporation of the Synaptix^®^ Digital Surgery Platform, which provides a real-time system information overlay displaying essential data such as instrument status, motion scaling, and recovery details, with the Symani^®^ Surgical System for microvascular anastomosis. Three surgeons (plastic surgery, neurosurgery, and head and neck) performed 1–2 mm synthetic vessel anastomoses using Symani alone and Symani with Synaptix. Metrics included Anastomosis Time, Teleoperation Exits, Re-Entry Time, Re-Entry Attempts, Assistant Use, and Exit Reason Match (alignment between system-logged and surgeon-identified explanations for each teleoperation exit event). Compared with Symani alone, mean Anastomosis Time was numerically shorter with Symani + Synaptix (20% reduction; 24.2 ± 7.2 vs. 19.4 ± 5.2 min), though this difference did not reach statistical significance (*n* = 3). Teleoperation Exits decreased by 44% (4.5 ± 0.5 vs. 2.5 ± 0.71). Re-Entry Time was reduced by 50% (0.38 ± 0.13 vs. 0.19 ± 0.01 min), and Re-Entry Attempts decreased by approximately 10%. Assistant Use decreased from 15% to 0%. Exit Reason Match was 75% when Synaptix was used with Symani compared to 26% when Symani was used without Synaptix. All surgeons reported that Synaptix improved visualization, reduced fatigue, and increased autonomy. Although performance gains varied by surgeon and vessel size, each demonstrated measurable benefit. These findings indicate that real-time digital assistance may streamline workflow, enhance performance across expertise levels, and facilitate broader adoption of robotic open microsurgery.

**Supplementary Information:**

The online version contains supplementary material available at 10.1007/s11701-026-03347-z.

## Introduction

Microsurgery and supermicrosurgery demand extreme precision, sustained concentration, and physical endurance. Even with advances in instrumentation, surgeons continue to face challenges with visualization, a precision gap, workflow efficiency, and physical fatigue, particularly when working with vessels ≤ 1 mm in diameter [1]. These limitations contribute to longer operative times and variability in outcomes, highlighting the need for solutions that both extend the technical capabilities of surgeons and optimize overall workflow.

Robotic platforms have expanded the technical possibilities of microsurgery by combining tremor reduction, motion scaling, and wristed micro-instruments [1–3], enabling precise vascular anastomoses at scales that surpass manual limits. Early feasibility studies have demonstrated that robotic-assisted microsurgery is safe and effective in confined anatomical spaces [2–7]. However, robotic procedures also introduce unique challenges, including the need for additional training [2] and workflow modifications [4, 8].

Digital assistance platforms have emerged as a promising strategy to enhance visualization, provide real-time workflow guidance, and minimize interruptions from unintentional exits [1, 2]. Such technologies have shown value in other surgical domains, however, there remains a critical gap in open microsurgery. Currently, no validated solutions exist that provide real-time, surgeon-centered digital assistance tailored to the demands of microvascular anastomosis at the 1–2 mm scale or smaller [2, 3, 8]. Addressing this gap could not only improve operative efficiency and visualization but also accelerate adoption of robotic systems in microsurgery.

The objective of this study was to evaluate the effect of the Synaptix Digital Surgery Platform, used with the Symani Surgical System, on surgical efficiency, error recognition, and surgeon experience during robotic-assisted vascular anastomoses on 1-mm and 2-mm synthetic vessels.

## Methods

### Study overview

This user study was designed to evaluate the effect of the Synaptix Digital Surgery Platform, integrated with the Symani Surgical System (both from MMI, Jacksonville, FL, USA), on microsurgical performance. All procedures were conducted at the MMI demonstration facility in Jacksonville, FL, USA.

MMI provided the Symani Surgical System, the Synaptix platform, and the Mitaka HawkSight 4K3D Video Microscope (Mitaka Kohki Co., Ltd., Mitaka-shi, Tokyo, Japan), which was used in both Symani-only and Symani + Synaptix procedures. The sponsor was also responsible for system setup and providing an overview of the Symani Surgical System and Synaptix. All data were independently verified for accuracy and quality assurance by designated observers.

Three experienced surgeons participated in the study, representing plastic surgery, neurosurgery, and head and neck surgery. None had prior clinical experience with or certification in the Symani Surgical System. Before study initiation, each was provided an overview of Symani that included demonstration sessions, supervised practice on synthetic vessels, and dedicated instruction on Synaptix workstation functionality.

### Synaptix overview

The Symani Surgical System is a robotic platform designed for open microsurgery, featuring motion scaling, tremor filtration, and wristed micro-instruments to enhance dexterity and precision in submillimeter procedures [1, 2] (Online Resource 1). Synaptix is an integrated digital assistance workstation designed to deliver an optimized experience for the entire clinical team during Symani robotic microsurgery by providing high-definition three-dimensional (3D) visualization of the operative field. It features a real-time system information overlay that displays essential data such as instrument status, motion scaling, and recovery details. The platform includes a visual alignment helper that provides on-screen cues to guide optimal controller positioning, as well as proactive alerts for motion and instrument/wrist limit thresholds to prevent teleoperation exits. Additionally, Synaptix supports intraprocedural image capture, including 2D/3D video recording, photo capture, and efficient storage management.

### Study design

Each surgeon completed four end-to-end synthetic vessel anastomoses in a fixed sequence. This included a 2-mm and 1-mm procedure performed with the Symani Surgical System alone, followed by a 2-mm and 1-mm procedure performed with Symani integrated with Synaptix, yielding 12 anastomoses in total. Surgeons placed six stitches per anastomosis using black nylon monofilament sutures (9−0 or 10−0). Although no formal quality scoring was performed, all anastomoses were independently observed and verified for accuracy and completeness by designated observers. All cases were performed under standardized conditions using the Mitaka HawkSight 4K3D Video Microscope. During Symani + Synaptix procedures, Synaptix provided real-time augmented visual guidance, integrated 3D visualization, and workflow assistance tools designed to reduce interruptions and improve task efficiency. Synthetic vessels were prepared and mounted by delegated technical staff provided by MMI before each procedure.

### Outcome measures

Primary and secondary outcomes included Anastomosis Completion Time, Number of Unintentional Teleoperations Exits, Duration of Exits (re-entry time), Number of Re-Entry Attempts, Need for Surgical Assistance, and Exit Reason Match (System vs. Surgeon). Definitions for all outcome metrics are provided in Table 1. Examples of on-screen overlays displaying system status, surgical status, and error-correction guidance during a procedure are available in Online Resource 2. For cases when Synaptix was not used, the observer asked the surgeon for the reason for teleoperation exit and compared his response to the information provided on the Symani Surgical System. All time-based measures (Completion Time and Exit Duration) are reported in minutes; fractional values < 1 therefore represent durations shorter than one minute. After each procedure, surgeons completed a structured questionnaire evaluating ergonomic motion guidance, visualization quality, perceived efficiency, mental workload, and overall user experience.

**Table 1:** Definitions of outcome metrics

Metric	Definition
Anastomosis Completion Time (minutes)	The total time, measured in minutes, required for the surgeon to complete the anastomosis procedure from start to finish.
Number of Unintentional Teleoperations Exits^a^	The count of instances where the robotic system disengages the surgeon from teleoperation due to safety or motion limit thresholds.
Duration of Exits (Re-Entry Time)	The time in minutes between a surgeon’s unintentional exit from teleoperation and their return.
Number of Re-Entry Attempts	The number of times the surgeon attempts to re-enter teleoperation after an unintentional exit before successfully resuming control.
Need for Surgical Assistance (Assistant Use)	Indicates whether a staff surgical assistant was required to intervene during the procedure.
Exit Reason Match (System vs. Surgeon)	The degree of agreement between the system’s logged reason for a teleoperation exit and the surgeon’s perception of the cause.
Anastomosis Completion Time (minutes)	The total time, measured in minutes, required for the surgeon to complete the anastomosis procedure from start to finish.
Number of Unintentional Teleoperations Exits	The count of instances where the robotic system disengages the surgeon from teleoperation due to safety or motion-limit thresholds.

^a^System-defined reasons for unintentional teleoperation exits were wrist limit exceeded, workspace limit exceeded, roll limit exceeded, instrument interference, speed limit exceeded, and tracking faults

### Statistical analysis

Surgeon feedback was recorded using a questionnaire form and Excel sheets for each surgeon and independently reviewed for accuracy. Data were analyzed to compare performance between Symani alone and Symani + Synaptix. Continuous variables (e.g., Anastomosis Time) were reported as mean ± standard deviation. Relative percentage change was calculated as (Symani – Synaptix)/Symani × 100. For binary outcomes (e.g., Assistant Use, Exit Reason Match), where baseline values could be far from 100%, results are reported as absolute percentage-point (pp) changes rather than relative percentages. Statistical analyses were descriptive due to the small sample size (*n* = 3 surgeons).

Paired comparisons were performed using Welch’s paired t-tests where data were available for all surgeons. Some workflow metrics were not applicable for one surgeon because no teleoperation exits occurred, preventing paired statistical testing. Accordingly, combined 1- and 2-mm values and metrics with non-applicable data are reported descriptively as mean ± SD without inferential statistics. For workflow metrics related to teleoperation exits (Re-Entry Time, Re-Entry Attempts, Assistant Use, and Exit Reason Match), values were analyzed only for cases in which an exit occurred; metrics were marked as not applicable when no exit was triggered.

Analyses were conducted using JASP 0.95.1.

## Results

### Surgeon-level time to complete anastomosis task

**Fig. 1:**
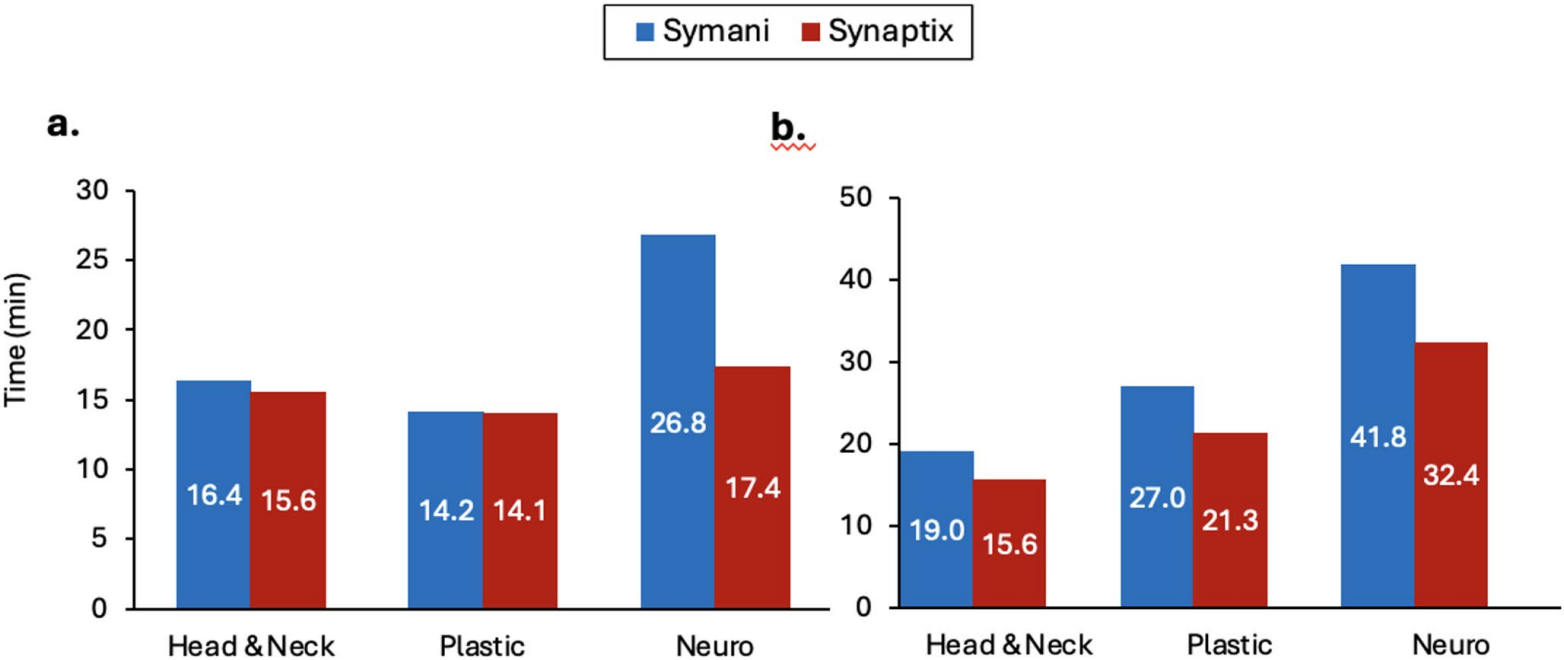
Anastomosis Completion Time (min) for 2 mm (**a**) or 1 mm (**b**) vessel models by surgeon (head & neck, plastic, neurosurgery) under two conditions: Symani Surgical System alone (blue) and Symani + Synaptix Digital Surgery Platform (red). Each bar represents a single anastomosis performed by an individual surgeon under the specified condition; no averaging or statistical comparisons were applied. All anastomosis-time data are complete for all surgeons and vessel sizes

Anastomosis Completion Times varied considerably across surgeons, vessel sizes, and use of the Synaptix Digital Surgery Platform in combination with the Symani Surgical System (Fig. 1; Table 2). To provide an integrated view, summary performance metrics are presented in Table 3, with full case-level data available in Table 2.

**Table 2:** Anastomosis completion times by surgeon, vessel size, and use of synaptix digital assistance

Surgeon	Vessel Size	Symani (min)	Synaptix (min)	Absolute Difference (min)	Relative Change %
Head & Neck	2 mm	16.4	15.6	0.8	5%
Head & Neck	1 mm	19.0	15.6	3.4	18%
Plastic Surgeon	2 mm	14.2	14.1	0.1	0.7%
Plastic Surgeon	1 mm	27.0	21.3	5.7	21%
Neurosurgeon	2 mm	26.8	17.4	9.4	35%
Neurosurgeon	1 mm	41.8	32.4	9.4	22%

Values represent the recorded time (in minutes) required to complete each anastomosis for individual surgeons performing microvascular suturing using the Symani Surgical System alone versus Symani combined with the Synaptix Digital Surgery Platform. Each entry reflects one observed case (*n* = 1) per surgeon and vessel size; no averaging across repeated trials was performed. Data are complete for Anastomosis Completion Times across all surgeons and vessel sizes

**Table 3:** Performance metrics for symani surgical system and symani + synaptix digital surgery platform

Metric	Symani mean ± SD	Synaptix mean ± SD	Absolute Difference	Relative Change (%/pp)	*p*-value
Anastomosis Completion Time (min; 2 mm)	19.1 ± 6.8	15.6 ± 1.7	3.5	18%	0.471
Anastomosis Completion Time (min; 1 mm)	29.3 ± 11.6	23.1 ± 8.6	6.2	21%	0.501
Anastomosis Completion Time (unweighted mean)	24.2 ± 7.2	19.4 ± 5.2	4.8	20%	–
Teleoperation Exits (count; 2 mm)	4.0 ± 4.0	1.7 ± 1.2	2.3	58%	0.296
Teleoperation Exits (count; 1 mm)	5.0 ± 3.6	3.0 ± 2.6	2.0	40%	0.329
Teleoperation Exits (combined count)	4.5 ± 0.5	2.5 ± 0.7	2.0	44%	–
Re-Entry Time (min)^a^	0.38 ± 0.13	0.19 ± 0.01	0.19	50%	–
Re-Entry Attempts (#; 2 mm)^a^	0.70 ± 0.57	1.00 ± 0.00	0.30	43%	0.504
Re-Entry Attempts (#; 1 mm)^a^	1.24 ± 0.21	0.72 ± 0.58	0.52	42%	0.158
Assistant Use (%; 2 mm)^a^	4.3 ± 7.51%	0.0 ± 0.0	4.3 pp	–	0.423
Assistant Use (%; 1 mm)^a^	23.7 ± 29.7%	0.0 ± 0.0	23.7 pp	–	0.302
Exit Reason Match (System vs. Surgeon)	26.0 ± 26.7	75.0 ± 43.3	51.0 pp	–	–

Performance metrics comparing the Symani Surgical System alone versus Symani with the Synaptix Digital Surgery Platform. Values are presented as means ± SD across surgeons. Metrics are reported separately for 2-mm and 1-mm vessels, with an additional descriptive row showing the unweighted mean of both conditions. Absolute differences are Symani minus Synaptix. Relative % change is calculated relative to Symani performance, except for Assistant Use and Exit Reason Match, which report absolute percentage-point (pp) differences. p-values were derived from Welch’s paired t-tests across surgeons; no p-values are reported for combined (2-mm + 1-mm) rows, as these represent descriptive aggregates only. Exit Reason Match is reported as a single combined value because the metric is only defined when a teleoperation exit occurs; several surgeon–vessel combinations had no exits, making stratification by 1-mm versus 2-mm not applicable

^a^For the plastic surgeon (Symani 2-mm and Synaptix 1-mm cases), teleoperation was never exited; therefore, Re-Entry Time, Re-Entry Attempts, and Assistant Use were marked as not applicable and excluded from those specific calculations; Exit Reason Matching was also not applicable

At the individual surgeon level, integration of Synaptix was generally associated with reduced completion times compared to use of Symani alone, with the magnitude varying by surgeon and vessel size (Fig. 1; Table 1). The greatest difference in Anastomosis Completion Times was observed for the neurosurgeon, with times 35% shorter in the Synaptix condition for the 2-mm vessel (26.8 vs. 17.4 min) and 22% shorter for the 1-mm vessel (41.8 vs. 32.4 min). Synaptix produced the greatest gains for the neurosurgeon, reducing Anastomosis Completion Times by 35% (26.8 to 17.4 min) for the 2-mm vessel and 22% (41.8 to 32.4 min) for the 1-mm vessel. The head and neck surgeon achieved more modest improvements, especially for the 2-mm vessels, with reductions of 5% (16.4 to 15.6 min) for the 2-mm vessel and 18% (19.0 to 15.6 min) for the 1-mm vessel. The plastic surgeon, who was an experienced microsurgeon, showed essentially unchanged times at 2-mm but recorded improvement of 21% (14.2 vs. 14.1 min) for the 1-mm vessel.

### Group level anastomosis performance across vessel sizes

**Fig. 2:**
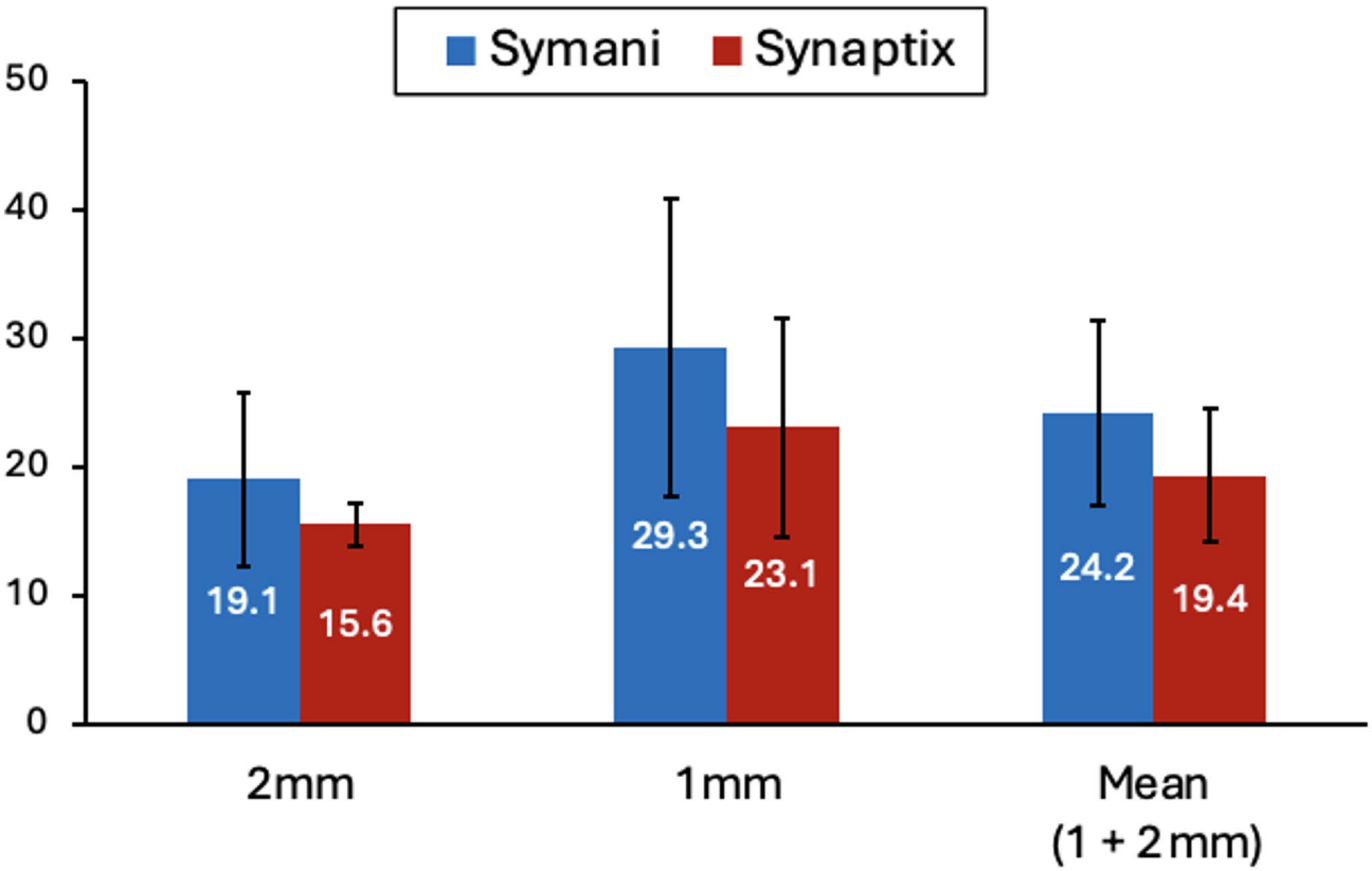
Anastomosis Completion Time for 2 mm, 1 mm, and pooled (combined average of 1- and 2-mm) vessel models under two conditions: Symani Surgical System alone (blue) and Symani + Synaptix Digital Surgery Platform (red). Each bar represents the mean across three surgeons, with error bars indicating the standard deviation (SD). For pooled (1- and 2-mm) values, SD was calculated from the combined set of six surgeon-level data points. Paired t-test results: 2 mm, *p* = 0.471; 1 mm, *p* = 0.501. No p-value is reported for the pooled values, as these reflect aggregated data rather than paired comparisons

Given the substantial variation across surgeons and vessel sizes, pooled means are presented for descriptive purposes only and should be interpreted cautiously as they mask meaningful individual-level differences. When aggregated across surgeons, use of Synaptix was associated with shorter mean Anastomosis Completion Times compared with Symani alone (Fig. 2; Table 3). For 2-mm vessels, mean time was numerically shorter in the Synaptix condition by 18% (19.1 ± 6.8 vs. 15.6 ± 1.7 min), though this did not reach statistical significance (*p* = 0.471). For 1-mm vessels, a similar pattern was observed, with mean time 21% shorter in the Synaptix condition (29.3 ± 11.6 vs. 23.1 ± 8.6 min; *p* = 0.501). When results from both vessel sizes were combined, mean Anastomosis Completion Time was 20% shorter in the Synaptix condition (24.2 ± 7.2 vs. 19.4 ± 5.2 min).

### Primary workflow efficiency indicators

**Fig. 3:**
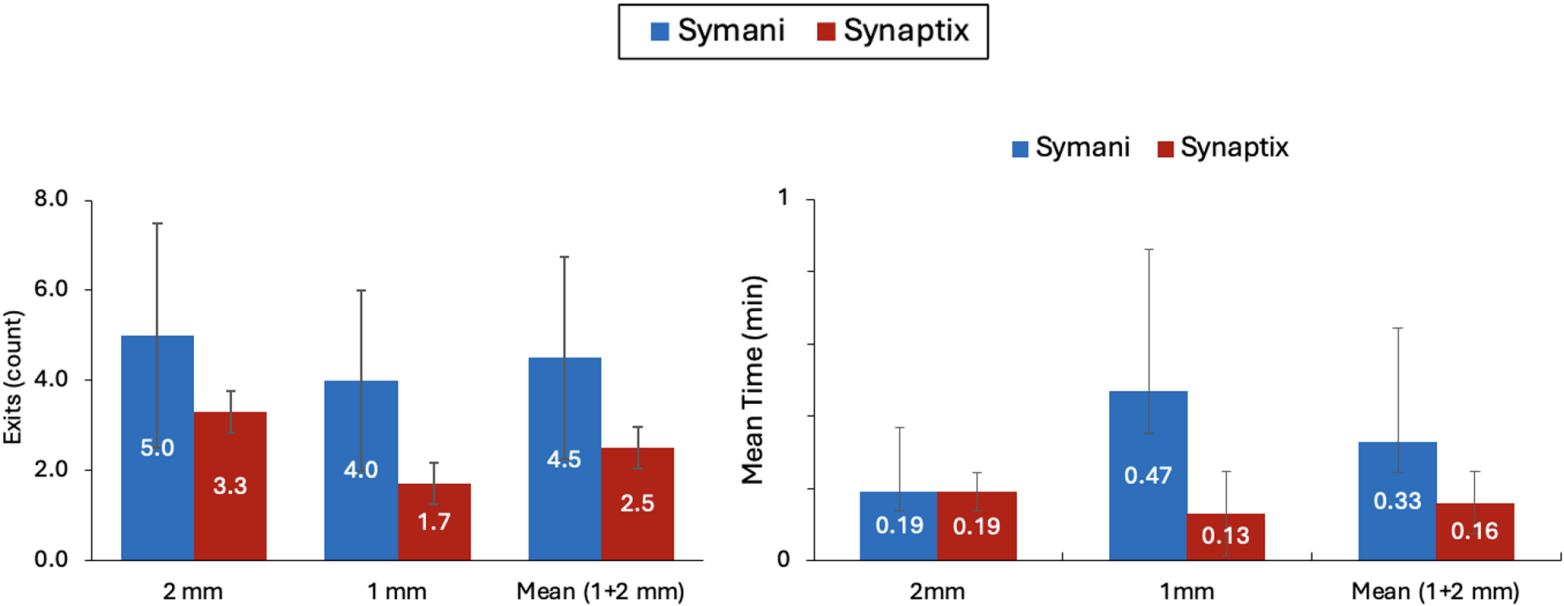
Number of Teleoperation Exits (**a**) and Re-Entry Time (**b**) for 2-mm, 1-mm, and pooled (combined average of 1- and 2-mm) vessel models under two conditions: Symani Surgical System alone (blue) and Symani + Synaptix Digital Surgery Platform (red). Each bar represents the mean across three surgeons, with error bars indicating the standard deviation (SD). For pooled 1- and 2-mm values, SD was calculated from the combined set of six surgeon-level data points. Teleoperation Exits represent discrete count data; variability is reported using mean ± SD for consistency across metrics but should be interpreted cautiously given the small sample size (*n* = 3). No p-values are shown for these metrics because missing data in some conditions precluded complete paired comparisons across all three surgeons. Values are therefore presented descriptively as mean ± SD

Synaptix use was associated with improvements in workflow efficiency metrics. The mean Number of Teleoperation Exits, which are instances where the system automatically disengaged the surgeon from teleoperation because of motion-limit thresholds, was evaluated across vessel sizes. Overall, Synaptix reduced the mean Number of Teleoperation Exits by 44% (4.5 ± 0.5 with Symani compared with 2.5 ± 0.7 with Synaptix; Fig. 3; Table 3). For Re-Entry Attempts, Assistant Use, and Exit Reason Match, some values were not applicable for the plastic surgeon in the Symani 2-mm and Synaptix 1-mm cases, as teleoperation was never exited; in these cases, the surgeon required no Re-Entry Attempts or Assistant Use, and Exit Reason Matching could not be assessed.

Re-Entry Time after a system-forced exit was reduced by 32% for 2 mm procedures performed by the head and neck surgeon (0.28 vs. 0.19 min) but increased by 14% for the neurosurgeon (0.22 vs. 0.25 min). In 1-mm procedures, Synaptix reduced Re-Entry Time by 62% for the head and neck surgeon (0.47 vs. 0.18 min) and by 76% for the neurosurgeon (0.92 vs. 0.22 min). When pooled across vessel sizes, Synaptix reduced Re-Entry Time by half (0.38 ± 0.13 vs. 0.19 ± 0.01 min), consistent with the individual-level trends.

Synaptix also reduced the mean Number of Re-Entry Attempts for both vessel sizes: by 43% for 2-mm procedures (0.70 ± 0.57 vs. 1.00 ± 0.00) and by 42% for 1-mm procedures (1.24 ± 0.21 vs. 0.72 ± 0.58) (Table 3).

### Workflow support and predictability

Assistant Use was defined as the proportion of cases where a staff surgical assistant was required to intervene to reposition instruments, manage exits, or troubleshoot. Higher assistant involvement reflects reduced surgeon autonomy and diminished workflow efficiency. Assistant Use occurred in 4.3% and 23.7% of 2- and 1-mm vessel cases, respectively, but was reduced to 0% with Synaptix, indicating complete elimination of Assistant Use.

Exit Reason Match (System vs. Surgeon) was defined as the degree of agreement between the system’s logged reason for a teleoperation exit and the surgeon’s perception of the cause. High agreement indicates transparent, predictable system behavior, which reduces cognitive load and enables the surgeon to return to the robotic procedure after workflow disruption. Agreement between the system-logged and surgeon-perceived reasons for Teleoperation Exits was 75% when Synaptix was used compared with 26% when using Symani alone.

### Surgeon-reported feedback

In addition to objective performance metrics, surgeon-reported experience was assessed through a structured survey to capture perceptions of Synaptix for real-world use. All three participating surgeons completed the structured portion of the survey, which included a series of statements rated for agreement. Responses showed universal agreement across all questions, including reports of improved efficiency, enhanced visual clarity, reduced operative time, decreased fatigue, and greater confidence during Symani use with Synaptix.

## Discussion

Robotic systems have expanded the possibilities of microsurgery, enabling highly precise vascular anastomoses at submillimeter scales [4, 8]. Early studies with robotic platforms demonstrated the feasibility of robotic-assisted microsurgery in small, technically challenging operative fields where visualization and maneuverability are limited [9, 10]. The Symani Surgical System, designed for open microsurgical procedures, uses wristed instruments to provide dexterity and precision beyond conventional tools, making 1-mm anastomoses technically feasible [8, 11]. While other robotic systems have controllers that are more mechanically restricted by attachment to jointed arms, Symani employs untethered controllers with a user interface that much more closely simulates natural arm motion and instrument use. Surgeons move these controllers within a defined workspace with motions translated to the operative site. Early in training, some surgeons may stray beyond the defined workspace, which results in an exit from teleoperation. By providing alerts when controllers move outside the working area and overlaying system status on the operative field, Synaptix facilitates rapid return to teleoperations.

This study demonstrates that integration of the Synaptix Digital Surgery Platform with the Symani Surgical System was associated with measurable improvements in operative efficiency and workflow continuity during microvascular anastomosis in synthetic vessel models. Synaptix, which received FDA clearance in September 2025 [12], offers features such as a 3D operating field display, system status overlays, visual alignment helpers, and workspace/motion-limit indicators. These tools work alongside Symani’s wristed instruments, tremor reduction, and motion scaling to reduce workflow interruptions, enhance autonomy, and improve procedural efficiency and precision.

While commercially available navigation and robotic systems have demonstrated success in orthopedic and thoracic applications [13–15], Synaptix represents one of the first digital platforms built for open soft-tissue microsurgery. Unlike systems that primarily emphasize precision guidance, Synaptix integrates enhanced visualization of the operative field and system status, real-time data overlays, and workflow management tools into robotic consoles, bringing digital surgery into a domain not typically served by these technologies. In contrast to prior studies, the present work directly evaluates the impact of Synaptix compared to Symani alone, enabling a controlled assessment of its effect on workflow efficiency and surgeon experience.

The participating surgeons had varied backgrounds in microsurgery and robotics, which shaped both baseline performance and perceived benefit of Synaptix. None had formal clinical experience or certification with the Symani Surgical System, though all completed informal demonstration sessions. The plastic surgeon had the most extensive microsurgical background, the head and neck surgeon had moderate experience, and the neurosurgeon reported the least familiarity with microsurgical techniques. These differences provide important context: even the most experienced microsurgeon demonstrated meaningful performance gains in technically demanding 1-mm vessels, indicating that digital assistance can enhance performance across different levels of expertise. Similarly, although the neurosurgeon exhibited mixed responses depending on vessel size, the substantial improvement in 1-mm tasks aligns with the observed benefits across other workflow metrics. By supporting surgeons across this spectrum, Synaptix may help those without formal microsurgical training perform delicate tasks, effectively lowering barriers to adoption of robotic microsurgery.

The pattern of individual-level results raises the possibility that the benefit of digital assistance may vary according to surgeon background and vessel size. Among the three participants, improvements in anastomosis time were observed across both vessel diameters, although the magnitude of improvement varied between individuals. This variability suggests that real-time digital guidance may offer differing levels of benefit depending on baseline technical familiarity with microsurgical tasks or procedural complexity. For example, one participant demonstrated substantial improvements across both vessel sizes, whereas another showed minimal change for 2-mm vessels, which may reflect a ceiling effect when technical steps are already highly optimized. Notably, measurable improvement was still observed for 1-mm vessels even among the most experienced participant, suggesting that digital assistance may remain valuable when addressing smaller, technically demanding targets. These observations should be considered hypothesis-generating and warrant future investigation in larger, stratified cohorts to better understand how surgeon background and vessel size may influence the impact of digital assistance.

Looking ahead, as the portfolio of robotic tools expands beyond anastomosis (e.g., energy instrumentation and FDA-cleared dedicated wristed dissection instruments [16]), Synaptix could serve as a critical assistance platform, helping surgeons integrate new capabilities into their workflows and enabling more microsurgical procedures to be performed robotically.

A potential learning effect across the session should also be considered, as surgeons became progressively more familiar with the Symani platform over successive cases. While features of Synaptix provide a plausible explanation for the observed efficiency gains, the contribution of task familiarization cannot be fully excluded. Future studies using counterbalanced or crossover designs may help isolate platform-specific effects from within-session learning. Notably, Synaptix was also associated with greater workflow stability, including fewer teleoperation exits, shorter re-entry delays, and reduced assistant involvement.

Motion-limit indicators and the visual alignment helper likely reduced teleoperation exits, while system overlays enhanced visualization of the operative field and supported efficient recovery when exits occurred [7]. Notably, these improvements occurred without altering how surgeon inputs were interpreted or how the system responded during teleoperation, and agreement between system-logged and surgeon-perceived exit reasons was preserved, supporting stable and predictable workflow recovery across surgeons.

Surgeon-reported experience aligned with the reported workflow-related gains. There was agreement on Synaptix’s performance and benefits, including sufficient visual clarity for all tasks, improved efficiency and autonomy owing to real-time system status displays, and reduction in operative time as well as physical and cognitive workload. Surgeons noted that the visual alignment helper improved controller positioning, motion and workspace limit indicators reduced unintended teleoperation exits, and Synaptix’s media capture tools were effective for intra-procedural documentation as well as post-procedure analysis and training. This subjective feedback parallels the objective efficiency metrics, reinforcing the interpretation that reduced workflow interruptions contribute meaningfully to perceived autonomy and confidence during robotic microsurgery.

In the broader context, prior preclinical studies established the feasibility of robotic microvascular anastomosis using wristed instruments [4, 11, 17], but few have systematically evaluated the role of digital overlays and workflow tools. Synaptix is designed to integrate visual guidance and real-time data into the operative field, enabling surgeons to access essential information without shifting focus to external monitors. This integration may provide a more seamless operative experience, which could enhance performance and facilitate long-term adoption in clinical practice.

This study has several limitations. The small number of participating surgeons reduced statistical power, and some workflow metrics were unavailable for one participant due to the absence of teleoperation exits, limiting the ability to conduct paired statistical testing. Accordingly, anastomosis time findings should be interpreted as descriptive numerical trends rather than statistically demonstrated effects, and the sample size was insufficient to power inferential conclusions for most metrics. Further, the use of synthetic vessels limit generalizability, and validation in clinical cases is needed. A fundamental limitation of this study is the fixed task sequence, in which all surgeons performed Symani-alone anastomoses before Symani + Synaptix anastomoses. Because none of the surgeons had prior clinical experience with Symani, the observed performance differences in the Synaptix condition likely reflect a combination of platform benefit and a within-session warm-up effect. The fixed sequence prevents causal attribution of the observed gains to Synaptix alone, and the magnitude of the learning curve contribution cannot be quantified from this design. A counterbalanced or crossover design would be required to isolate platform-specific effects from those attributable to increasing familiarity with the system. Additionally, no formal anastomosis quality metrics were collected, such as leak testing or blinded suture placement scoring; efficiency gains therefore cannot be fully interpreted without corresponding quality data, and future studies should incorporate objective quality assessments as primary or co-primary outcomes. Finally, surgeons were not blinded to the intervention (use of Synaptix), so it is impossible to rule out a Hawthorne effect on performance due to the presence or absence of the intervention.

Future directions include further refinement of digital assistance tools such as Synaptix to better support microsurgical practice. Additional mechanisms to streamline workflow, potentially including automated tasks that enhance efficiency and standardization, warrant investigation. Enhancements that aid intraoperative decision-making, such as more integrated intraoperative data visualization or context-specific guidance, also represent promising areas for development. In the longer term, the ability to review procedural metrics and performance trends may inform training and operative planning. Prospective studies should incorporate objective anastomosis quality metrics, including leak testing and blinded expert suture placement scoring, as primary or co-primary outcomes alongside efficiency measures, to determine whether workflow improvements are achieved without compromise to anastomosis quality.

## Conclusions

This pilot study provides preliminary evidence that integrating the Synaptix Digital Surgery Platform with the Symani Surgical System is associated with improved microsurgical efficiency and workflow continuity compared to Symani alone. By providing real-time overlays, visual alignment aids and workspace and motion-limit indicators, Synaptix was associated with shortened anastomosis times, reduced eye strain, minimized workflow interruptions, and increased surgeon confidence while using Symani. These findings highlight the potential of digital assistance to streamline operative workflow, enhance surgeon autonomy, and support broader adoption of robotic approaches in open microsurgery. Future prospective studies using counterbalanced designs and larger, stratified cohorts will further define the role of digital assistance across surgeon backgrounds and vessel sizes.

## Supplementary Information

Below is the link to the electronic supplementary material.

Supplementary Material 1

## Data Availability

All data supporting the findings of this study are available within the paper and its Supplementary Information.
