# Supplementary material for: Real-time digital assistance improves surgeon efficiency and autonomy in robotic microvascular anastomosis: a pilot feasibility study
Source: J Robot Surg. 2026 Apr 2;20(1):400. doi: 10.1007/s11701-026-03347-z (PMC13046566; doi:10.1007/s11701-026-03347-z)
Supplement: Supplementary file 1 — Supplementary Material 1 [file 11701_2026_3347_MOESM1_ESM.docx]

**Real-Time Digital Assistance Improves Surgeon Efficiency and Autonomy in Robotic Microvascular Anastomosis**

**Journal of Surgical Robotics**

Jesse Selber, MD, Hari Jeyarajan, MD, Ricard Hanel, MD

*Affiliation*

Jesse Selber, MD, MPH, MHCM

Corewell Health William Beaumont University Hospital, (Department of Plastic & Reconstructive Surgery), Royal Oak, Michigan, USA

*Email of corresponding author:*

[jesse.selber@corewellhealth.org](mailto:jesse.selber@corewellhealth.org)


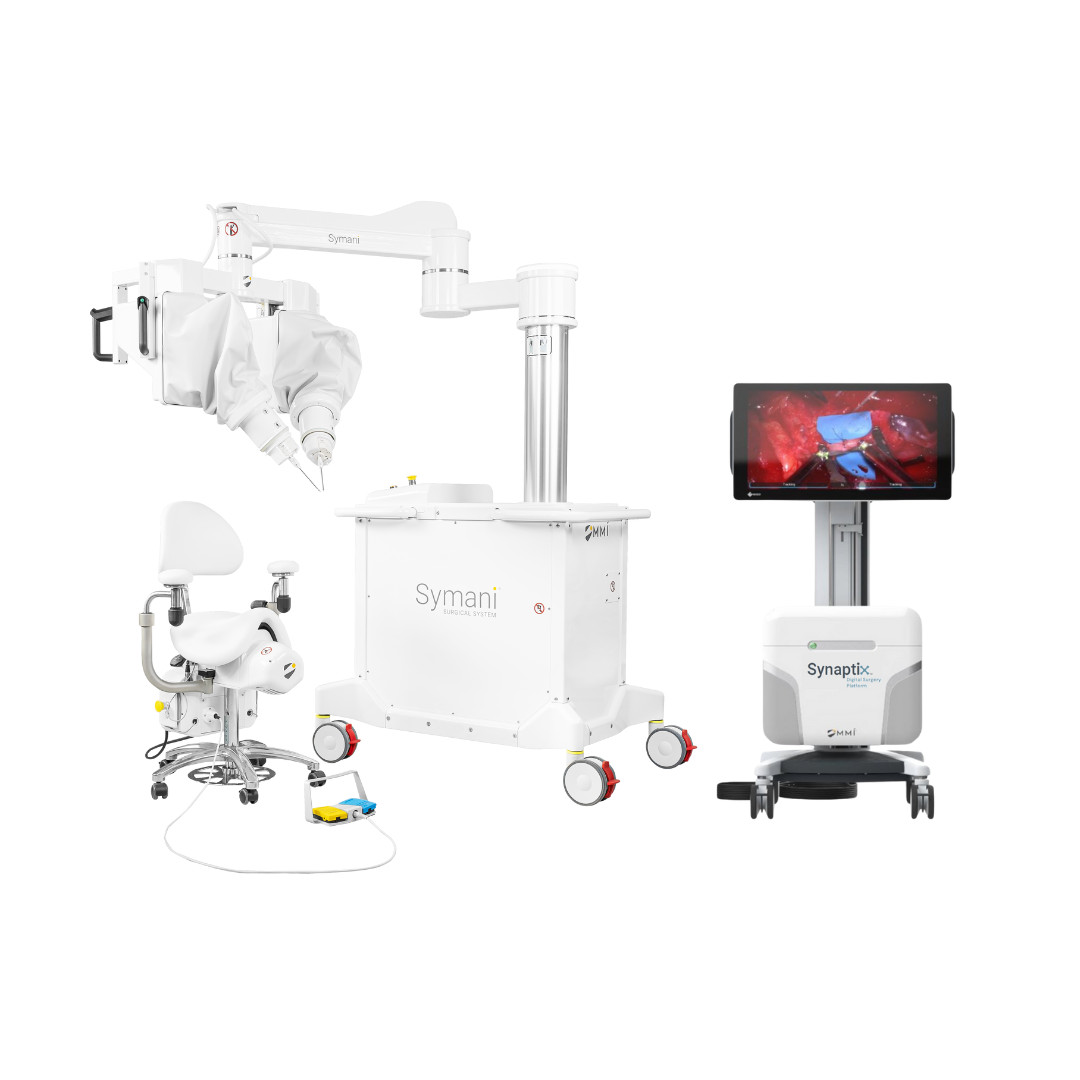


**Online Resource 1.** The Symani Surgical System (left), featuring wristed microinstruments and surgeon console, and the Synaptix Digital Surgery Platform (right), which provides integrated visualization and system-status displays.


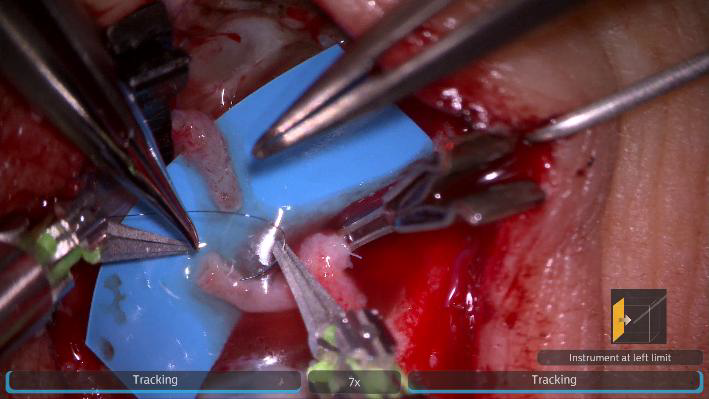

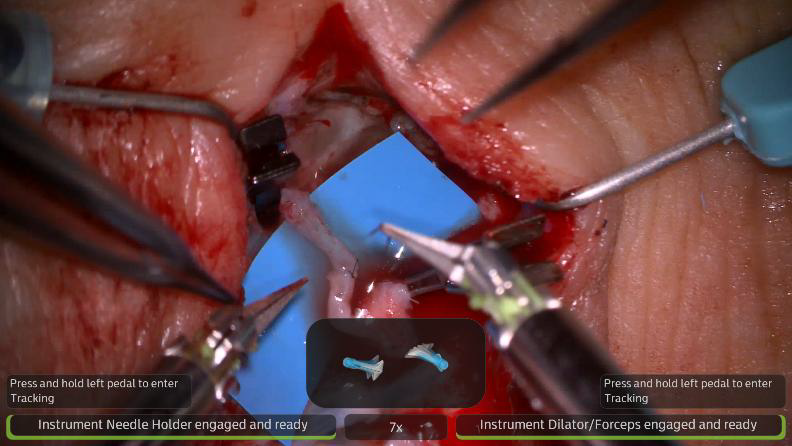

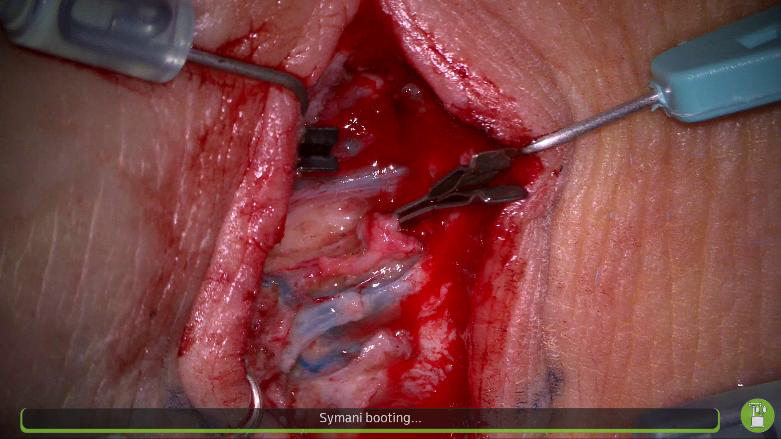

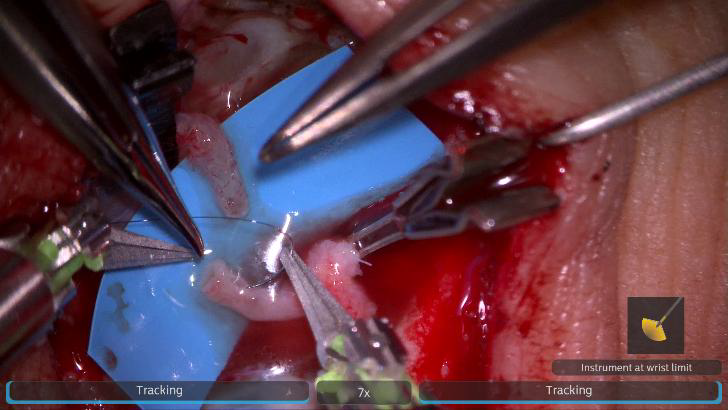


**a**

**b**

**c**

**d**

**Online Resource 2.** Examples of on-screen overlays displaying system status, surgical status, and error-correction guidance during a procedure.

(a) Start-up phase with system initialization message.

(b) Instrument engagement phase with the visual alignment widget displayed at the center of the field.

(c) Tracking phase with notification indicating the instrument has reached its left limit.

(d) Tracking phase with notification indicating the instrument has reached its wrist limit.
